# ResQ Family: Respiratory Syncytial Virus (RSV) Infection in Infants and Quality of Life of Families—Study Protocol of a Multi-Country Family Cohort Study

**DOI:** 10.3390/ijerph20115917

**Published:** 2023-05-23

**Authors:** Ilona Trautmannsberger, Sabina Bösl, Christina Tischer, Johanna Kostenzer, Silke Mader, Luc J. I. Zimmermann, the ResQ Family Study Group

**Affiliations:** 1 European Foundation for the Care of Newborn Infants (EFCNI), 81379 Munich, Germany; 2 Department of Health Security, Finnish Institute for Health and Welfare, FIN-70701 Kuopio, Finland; 3 Department of Paediatrics, Research School for Oncology and Reproduction, Maastricht UMC+, 6229 ER Maastricht, The Netherlands

**Keywords:** respiratory syncytial virus (RSV), quality of life, HRQoL, parents, infants, children, family, participation, cohort study

## Abstract

(1) Respiratory syncytial virus (RSV) infection in infants not only affects the child itself, but also their families. Nevertheless, information on the overall impact is scarce. A comprehensive caregiver-specific approach covering essential (health) dimensions and relevant stakeholders was initiated under the ResQ Family study conducted in Germany, France, Italy and Sweden. The primary objective is to investigate the health-related quality of life of parents and/or caregivers of children (<24 months) hospitalised for RSV. (2) Each participant completes an online questionnaire disseminated via social media and printed material in hospitals. Using the PedsQL^TM^ FIM as well as further self-designed questions, parent and patient characteristics as well as potential stressors and preventive factors are recorded at baseline and after six weeks. Multivariate regression models with health-related quality of life as main outcome parameter will be conducted. (3) The study is currently in the recruitment process. A full analysis will be performed once the data collection phase is complete. First results are to be expected in late 2023. (4) Publishing the results in the form of scientific papers but also non-scientific (information) material will help us raise awareness for RSV and the importance of prevention among healthcare professionals, patient representatives and decision-makers.

## 1. Introduction

Respiratory syncytial virus, or RSV, is a highly contagious virus causing lower respiratory tract infections (LRTI), particularly in very young children [1]. It commonly arises during the coldest months of the year; however, shifts were reported due to preventive measures related to the ongoing COVID-19 pandemic [2,3,4]. Each year, on a global scale, RSV leads to approximately 33 million cases of acute LRTI, resulting in more than three million hospitalisations in children under 5 years of age [5]. In the context of a European community setting, direct costs per admission can be estimated at EUR 9682.8 representing a high (socio)economic burden not only for healthcare payers (EUR 4587.9) but also from a societal perspective (EUR 5094.9) [6]. Although preterm infants and children with pre-existing comorbidities are at high risk for acquisition, nearly all infants will have been infected with RSV at least once by the age of two years, resulting in the hospitalisation of one in every 56 healthy term-born infants in high-income countries [1,7,8,9,10]. In the course of the infection, RSV often leads to bronchiolitis and pneumonia requiring (invasive) supportive care measures such as supplemental oxygen, fluid replacement, mechanical ventilation or intubation which might result in long-term complications, such as wheezing or impaired lung function later in childhood [11,12,13].

When the RSV infection takes a severe course, it might not only affect the infant’s health but also significantly impact the life of other family members. Illness and hospitalisation of children are often critical events leading to broad implications on everyday life resulting in feelings of stress and anxiety especially for the affected parents [14]. These conditions are part of the health-related quality of life (HRQoL) concept, a key health outcome indicator in research studies [15,16]. HRQoL is the subjective well-being or quality of life relative to one’s health or disease status and can be conceived as a multi-dimensional approach including emotions, mental health as well as problems in relation to daily activities, work productivity (loss) and family functioning [17,18,19]. In the context of RSV, various stressors associated with the child’s hospitalisation can, therefore, impose a significant burden on the entire family, leading to a wide range of detrimental effects on their daily life [20,21].

To date, previous studies investigated only isolated aspects of paediatric RSV infection in relation to the child’s and/or parental (health-)related quality of life, addressed respiratory diseases in general or did not use a caregiver-specific instrument [21,22,23,24]. However, some studies exploring aspects of the burden of respiratory infections in families were conducted. In 2021, Díez-Gandía and colleagues reported a mean HRQoL loss in a Spanish population of RSV-infected children and their parents of 38% during the first week of symptoms, using an ad hoc questionnaire over a period of 30 days [22]. In France, Lapillonne et al. showed that, in particular, the emotional, physical and daily organisational aspects of all family members were clearly impacted due to the infant’s hospitalisation for bronchiolitis [20,23]. Another study conducted in Sydney, Australia, evaluated an assessment tool to measure the quality of life of caregivers of children with influenza-like illnesses (Care-ILI-QoL) and showed significant decreased total QoL scores in parents with perceived very/extremely sick children [24]. Yet, the impact is not fully known, as there is, to our knowledge, no study available addressing the burden and impact on the entire family, often described as the “Greater Patient” with a caregiver-specific multi-national approach covering essential (health) dimensions and involving relevant stakeholders [25]. To fill existing knowledge gaps, address the public health burden and emphasize the caregiver perspective in this specific field of research, the ResQ Family study (ResQ Family: Impact of Respiratory Syncytial Virus hospitalisation on Quality of life of Families—a multi-country study) was initiated.

This study protocol provides the scientific basis for the ResQ Family project (http://www.resq-family.org, accessed on 10 March 2023). It defines the study design and study objectives, describes the recruitment of the study population and explains the procedures, evaluations and statistical analysis to be performed. The overarching aim of the ResQ Family study is to comprehensively investigate the HRQoL of parents and caregivers of children hospitalised due to RSV by applying a participatory research approach.

The following main hypotheses can be defined:

(a) An RSV infection with hospitalisation has an impact on the health-related quality of life (HRQoL) of affected parents and caregivers;
(b) The severity of an RSV infection with hospitalisation is associated with health-related quality of life (HRQoL) of parents and caregivers (over time);
(c) Socio-economic status, parental health literacy and support modify the association between the severity of an RSV infection with hospitalisation and health-related quality of life (HRQoL) of parents and caregivers.

## 2. Materials and Methods

### 2.1. Study Population and Design

The ResQ Family study runs from December 2021 until June 2024. Data are collected between September 2022 and May 2023, corresponding to a typical RSV season duration in countries of the northern hemisphere. The project was designed as a multi-country observational study in which parents and caregivers of children hospitalised for RSV were invited to share their experiences via an online questionnaire.

Ensuring geographical distribution across Europe (Northern, Central and Southern Europe), participants living in Germany, France, Italy and Sweden are enrolled in the study. All parents and caregivers whose children (i) are under 24 months of age, (ii) were diagnosed with RSV either by a confirmed test (e.g., ELISA/IFT/PCR from nasal secretion, sputum or throat swab) or a doctor’s diagnosis based on typical symptoms during the RSV season, (iii) were hospitalised no more than four weeks ago (calculated from the day of hospital admission to the day the questionnaire was completed) for at least 12 h due to the respective RSV infection are included in the study.

After completion of the baseline survey, study participants have the opportunity to either end with an anonymous one-time attendance or provide their e-mail address for follow-up participation six weeks later, examining the lasting impact (on the family) after (the child’s) hospitalisation due to RSV.

### 2.2. Participation and Involvement of Relevant Stakeholders

In general, and to ensure the involvement of relevant stakeholders, the ResQ Family project is accompanied by a multi- and transdisciplinary working group comprising the “External Scientific Advisory Board (ESAB)” composed of researchers specialised in the field of respiratory infectious diseases and/or quality of life and further parent and patient representatives as well as healthcare professionals as part of the “Project Expert Group (PEG)”. Each country is, thereby, represented by at least one parent (either as a representative of a national parents’ organisation or an affected caregiver) and one paediatrician or neonatologist experienced with RSV.

Initial ideas for the study design were developed interactively together with all members of the ESAB and PEG during a roundtable discussion in April 2022 and further refined in separate expert and parent meetings. In the following and through regular exchanges, the working group agreed on inclusion and exclusion criteria, gradually developed the questionnaire and generated recruitment materials.

Throughout the recruitment phase, continuous contact will be maintained to review and, if necessary, adapt the recruitment strategy.

### 2.3. Recruitment of Study Participants

Parents and caregivers can be approached during hospital stay, at or shortly after discharge of their child hospitalised for RSV. However, conducting the survey has to take place within four weeks following the first day the child was hospitalised. This time frame was defined taking into account the average length of stay of RSV-infected children in Europe (~2.7 days) as well as the underlying study conditions intending to ensure the recruitment of a maximum number of participants, while avoiding potential recall bias [26].

Participants are mainly recruited by receiving the link to the online survey via social media and newsletter channels of the European Foundation for the Care of Newborn Infants (EFCNI) and their network. In addition, parent organisations, healthcare professionals in hospitals, professional healthcare societies—especially those included in the ESAB and PEG—as well as further networks in Germany, France, Italy and Sweden are encouraged to support the promotion of the study. They are asked to either directly engage in in-person recruitment of possible participants by distributing study-specific printout materials to affected families or to spread the survey online via their network(s).

For this purpose, a multilingual recruitment toolkit was prepared and professionally designed. The toolkit provides an overview of the project communication and survey recruitment strategies in general and includes ready-to-use elements such as graphics and text templates for social media (Facebook, LinkedIn, Instagram, Twitter), mailings, and newsletter communication, as well as templates for printout materials (postcard, flyer/leaflet). To ensure suitability and comprehensibility for the target group, all recruitment and information material was developed and, subsequently, translated in collaboration with a skilled communications team as well as native-speaking parents and medical experts of all four participating countries.

To clarify open questions, to explain the application of the toolkit in more detail and to elucidate the possibility of distributing printout materials, additional briefing calls with experts (ESAB and PEG members) of every participating country were carried out. The toolkit was then made available in all language versions on EFCNI’s project-related website (http://www.resq-family.org, accessed on 10 March 2023) and distributed via e-mail to the expert network developed and involved in the study. In addition, printed postcards were sent to all interested parties who offered to distribute them to affected families in neonatal/paediatric wards in hospitals (e.g., together with the discharge letter) or corresponding networks.

### 2.4. Data Collection

Data for the baseline survey were collected via a self-report online questionnaire of 84 single- and multiple-choice closed questions and one open question. Besides socio-demographics of the participating parent or caregiver and the RSV-infected child, details of the child’s comorbidities as well as the child’s symptoms during RSV infection and hospitalisation are recorded. To assess the impact of the disease on HRQoL of affected families the standardised and caregiver-specific instrument PedsQL^TM^ Family Impact Module (PedsQL^TM^ FIM) as well as further self-designed questions based on existing instruments and literature in the respective field are used [27,28]. Completing the questionnaire requires approximately 20 min in total.

To evaluate changes over time, parents and caregivers who consented for future contact were invited by e-mail to take part in a follow-up six weeks after the baseline survey. A re-query of the PedsQL^TM^ FIM, further single- and multiple-choice closed questions and one open question give participants the opportunity to add more information about persistent symptoms or recovery, possible barriers and the long-term impact of the child’s RSV infection on the family in general. The time required to answer the total of 23 questions in the follow-up survey is estimated at 5–10 min.

All questions of the baseline and follow-up survey were initially set up in English, serving as a master version for the respective country languages. With the help of native speaking parent representatives and health care professionals of the PEG, questions which were not already part of the validated PedsQL^TM^ FIM were then translated into German, French, Italian and Swedish, ascertaining (linguistic) comprehensibility and accessibility to all study participants. To guarantee the expertise of both parents and doctors, each language was reviewed by at least one parent representative and one medical expert fluent in the corresponding language. By using a translation editor, all final language versions were incorporated into SurveyMonkey, an online survey tool which fulfils the requirements of the General Data Protection Regulation (GDPR) [29,30]. After the final set-up, native speakers pre-tested the questionnaire in all four languages.

### 2.5. Research Outcomes and Instruments

#### 2.5.1. Socio-Demographic Characteristics and Child’s Health Status

At baseline, different variables such as age, gender, highest level of education, civil status, (legal) relationship to the child, place of residence and living conditions are included. The presence of a history of prematurity, allergic diseases or other comorbidities as well as symptoms of the current RSV infection and the specific infant’s quality of life are measured using self-developed items adapted from questions by Díez-Gandía et al. [22]. Moreover, a severity index comprising the child’s RSV symptoms based on Leidy et al. will be calculated [21].

#### 2.5.2. Impact on Family/Caregiver

The PedsQL^TM^ FIM serves as the main parent-reported outcome measurement instrument of the ResQ Family study and is assessed at baseline as well as in the follow-up survey [27,28]. Other measurement instruments such as the Parenting Stress Index or the Caregiver Impact Questionnaire were not available in all required languages, were not caregiver-specific (e.g., SF-6D, EQ5D-5L or PROMIS), were disease-specific for bronchiolitis (Impact of Bronchiolitis Hospitalization Questionnaire) or were not yet validated (e.g., ad hoc 38 item-questionnaire developed by Díez-Gandía et al.) and, therefore, not used or in a modified form only [22,23,31,32,33,34,35]. The PedsQL^TM^ FIM consists of 36 items and was designed to measure the impact of paediatric acute and chronic health conditions on parents and the family with a recall period of one month [27,28,36]. Six parental self-reported dimensions are covered: physical, emotional, social and cognitive functioning, communication and worry [27]. The module also comprises items on parent-reported family daily activities and family relationships [27]. Reliability and validity were demonstrated in populations of chronically diseased children with, for example, cerebral palsy and birth defects (and their parents/caregivers) and were also successfully tested in a community sample [27,37]. The PedsQL^TM^ FIM was validated in all official languages of the four participating countries (Swedish, German, French, Italian) and allowed the calculation of a sum score [27].

Further self-developed items based on questions from Díez-Gandía and colleagues about parental worry or concern with regard to the child’s RSV symptoms were added [22]. To ensure the target group-specific relevance and completeness of the questionnaire, an expert interview with a mother of an infant recently hospitalised for RSV was conducted. Based on her feedback and insights from previous projects, further important aspects of parents’ personal feelings during and after the hospital stay were incorporated [38,39]. As a result, questions on the possibility to be present and interactive with the child receiving intensive care, loss of work productivity and additional barriers as well as parental health literacy and support were added to the survey. Three disease-independent screening questions additionally assess the general health literacy of parents/caregivers [40]. To investigate other possible unmet needs, feelings or coping strategies in relation to the RSV infection of their child and/or the hospitalisation, parents or caregiver were asked about their personal experiences in an open question.

A summary of all measurement instruments and measurement time points can be found in Table 1.

### 2.6. Statistical Analysis

The statistical evaluation will include descriptive, correlation and analytical analyses.

To measure the general impact of an RSV infection with hospitalisation on the HRQoL of affected parents and caregivers, sum scores of the PedsQL^TM^ FIM are compared to results from a community sample study [37]. Correlation analyses will be conducted between parental/caregiver quality of life and severity of the infant’s RSV infection, parental health literacy and supporting factors, work productivity (loss) and socio-demographic characteristics [21]. If variables are proven to be normally distributed, an independent sample t-test will be used; otherwise, a non-parametric alternative. A change over the observation period (t0—baseline survey until t1—follow-up study) in variables measured at both time points (see Table 1) will be calculated applying paired t-tests or by means of a non-parametric alternative (pre-post effects). With the help of multiple linear and logistic regression, it is anticipated to assess key determinates (potential stressors and preventive factors) associated with the HRQoL of affected parents and caregivers as main outcome parameter cross-sectionally and over time. The final models will be adjusted for relevant confounders such as socio-economic factors, RSV prophylaxis, comorbidities, prematurity or symptom status. Stratified and/or subgroup analysis (e.g., confirmed diagnosis vs. doctor’s diagnosis) will be performed to estimate subpopulations.

All statistical analyses will be performed using the statistical software R (R Core Team (2022), URL https://www.R-project.org, accessed on 10 March 2023) or SPSS (IBM SPSS Statistics for Windows, V.27–0, IBM Corp, Armonk, New York, NY, USA) with a significance level set at 5%. The evaluation of the open questions will be carried out by means of a structuring content analysis and with the support of an evaluation software (e.g., MAXQDA) [41,42].

### 2.7. Ethics and Dissemination

Research data collected from parents and caregivers will be stored locally based on established principles of data protection and security. The ResQ Family study was designed according to the principles of the Declaration of Helsinki and the need for ethical approval was waived by the Ethical Committee of the University of Maastricht in the Netherlands on 4 August 2022 (reference number METC 2022-3307) prior to the start of recruitment. As the Medical Research Involving Human Subjects Act (WMO) does not apply to the study, it was confirmed that an official approval was not required. After confirmation of ethical clearance, the study was registered at clinicaltrials.gov (ID: NCT05550545). Participation is voluntary at all times and the study participants are informed about the content and aim of the ResQ Family study before giving their consent. The results will be published in open access peer-reviewed journals and further disseminated through international conference contributions. Ultimately, the findings will be vividly prepared in the form of scientific and non-scientific information material and shared with policy makers, national and international parent organisations and the general public.

## 3. Results

The ResQ Family study is currently in the recruitment process which will take place from September 2022 until May 2023. Interim analyses will be carried out in the following months and a full analysis of the data will be conducted once the recruitment and data collection of the follow-up is completed. The results are planned to be published in an international peer-reviewed journal.

## 4. Discussion

By applying a participatory research approach and involving relevant stakeholders, the ResQ Family study intends to provide scientific evidence on the HRQoL of parents and caregivers of children hospitalised due to RSV in a comprehensive manner. It aims first to generate knowledge not only on the HRQoL of parents and children, but also to identify whether a variety of additional key determinants such as support or productivity loss are associated with this multi-faceted construct. Finally, the findings can contribute to minimise the public health burden of RSV and its consequences by identifying and promoting disease prevention and support structures such as parental health literacy.

Compared to other studies, which are often limited to one country, the ResQ Family project is multi-national in scope [20,22]. Including four European countries, higher rates of patient enrolment, a representativeness of the burden of RSV and its impact on affected families across Europe as well as the possibility for direct country comparison are expected. Moreover, with the PedsQL^TM^ FIM, an appropriate and validated instrument specifically designed for caregivers, the ResQ Family project is, to our knowledge, the first study using a caregiver-specific instrument to address HRQoL of parents/caregivers of children hospitalised due to RSV [20,21,22,24,27,28]. Further items were adapted based on existing instruments and published literature in the respective field. The continuous exchange with (affected) parents during the ResQ Family questionnaire development and translation into the respective country languages was crucial to cover all important aspects but also to ensure comprehensibility. Involving relevant stakeholders such as parents, researchers and medical experts right from the beginning of the study enables participatory research on equal terms and simultaneously guarantees a high level of medical and methodological expertise. A collaborative and transnational research approach further facilitates addressing previously unmet needs and will ultimately help us raise awareness for the importance of preventive measures for childhood RSV infections among healthcare professionals, patient representatives, decision-makers and the general public.

However, some limitations need to be depicted. The study has an observational family cohort study design and is, therefore, not able to reveal causal relationships. Direct effects cannot be estimated as the study lacks a control group. Nevertheless, and provided that sufficient participation in the follow-up study six weeks later is achieved, the study group is confident to be able to inform about changes of the HRQoL of affected families over time and in different European healthcare settings. Recruitment of participants is primarily conducted via social media channels and, thereby, follows a voluntary response procedure, possibly addressing a certain target group only to a certain extent. This could have a potential impact on the representativeness of the results and may lead to sampling bias. As this survey is a self-reported questionnaire, potential self-report biases due to factors such as social desirability can occur [43]. Another problem might arise in the diagnosis of children who are not officially tested for RSV. This could lead to an inclusion of children with a suspected but not confirmed RSV infection. Lastly, since the study exclusively considers hospitalised RSV patients, results cannot be fully transferred to outpatient cases.

## 5. Conclusions

The ResQ Family study will contribute to acquiring new knowledge on the burden of RSV and its multidimensional impact on parents and caregivers of infected children. The results can help to identify stressors and, on this basis, also preventive factors in order to counteract and minimise the negative consequences on HRQoL aspects for future affected families as well as healthcare providers and, eventually, the entire society. Ultimately, novel approaches for disease prevention and support structures will be offered and awareness on the global impact of RSV will be raised between policy makers, healthcare professionals and the general public.

## Figures and Tables

**Table 1:** Overview on domains/variables/instruments/time points

**Domains**	**Variables/Constructs**	**Score/Instrument and Data Source**	**Time Point**
Parental/family socio-demographic characteristics	Age, gender, highest level of education, civil status, (legal) relationship to the child		t0
Infants’ socio-demographic and health characteristics	Age, gender, place of residence, living conditions and circumstances, gestational age, birth weight, type of pregnancy, nutrition (before and during hospitalisation), history of allergic diseases, compliance with vaccination recommendations, RSV prophylaxis		t0
Infant’s comorbidities	Pre-existing health conditions		t0
Infant’s symptoms during RSV infection and hospitalisation	Place of first diagnosis, type of diagnosis (confirmed diagnosis vs. doctor’s diagnosis), symptoms during or after the RSV infection and hospitalisation, length of hospital stay, type and duration of symptoms, clinical manifestations, supportive care measures, place of treatment	Based on Díez-Gandía et al. [22]	t0, t1
Infant’s quality of life	General behaviour, sleeping behaviour, irritability, playing behaviour, exhaustion, attentiveness, demand for comfort	Based on Díez-Gandía et al. [22]	t0, t1
Worry/concern about child’s RSV infection/symptoms	Degree of overall worry, degree of worry about specific symptoms, feelings about child’s health status during hospitalisation	Based on Díez-Gandía et al. [22]	t0, t1
Presence with the child receiving special/intensive care	Permitted visits and visitors, frequency and duration of visits/presence, interaction with the child, involvement in child’s treatment	Based on Kostenzer et al. [38,39]	t0
Impact of child’s disease on parents/caregiver/family	Physical Functioning	PedsQL^TM^ Family Impact Module [27]	t0, t1
	Emotional Functioning		
	Social Functioning		
	Cognitive Functioning		
	Communication		
	Worry		
	Family daily activities		
	Family relationships		
Parent/caregiver general health status	Scale 1 (very bad)—10 (excellent)		t0, t1
Loss of work productivity	Current professional status, working hours, missed working hours, impact on job productivity, impact on leisure activities		t0, t1
Barriers visiting hospitalised child	Distance, time, additional costs		t0
Health Literacy	Awareness of disease complications/consequences, knowledge of preventive measures, comprehension of child’s infection and treatment, knowledge about support for mental health problems	Screening test for inadequate health literacy [40]	t0
Support	Degree of information on child’s health status during hospitalisation, channel and type of information, information on follow-up care and preventive measures to avoid re-infection, information on mental health support, type of mental health support		t0
Overall impact of RSV infection on parent/caregiver and family	Open question (additional feelings, potential coping strategies, enduring impact/problems)		t0, t1
Follow-up of child’s RSV infection	Medical check-up		t1

## Data Availability

Not applicable.
